# Electrospun Integrated Janus Nanofibers for Asynchronous Delivery of Finasteride and Tibetan Medicine

**DOI:** 10.3390/pharmaceutics18091160

**Published:** 2026-09-15

**Authors:** Qilin Wang, Tingyu Chen, Hailong Dou, Deng-Guang Yu

**Affiliations:** School of Health Science and Engineering, University of Shanghai for Science and Technology, Shanghai 200093, China; 243403174@st.usst.edu.cn (Q.W.); 243403094@st.usst.edu.cn (T.C.); 2335051410@st.usst.edu.cn (H.D.)

**Keywords:** Janus nanostructure, Tibetan medicine, tri-fluid electrospinning, co-shell solvent, asynchronous drug delivery

## Abstract

**Background**: Simultaneous controlled release of multiple therapeutics from a single dosage form is a major frontier in modern pharmaceutics and a key strategy for modernizing traditional Chinese medicine. **Methods**: Using finasteride (FIN) and a Jing-Zhu hybrid Tibetan (JZ) herbal medicine as model agents for prostatitis therapy, we developed a modified tri-fluid electrospinning process to fabricate drug-co-loaded Janus medicated nanofibers. Soluble polyvinylpyrrolidone (PVP) and insoluble ethylcellulose (EC) served as carrier matrices for the dual faces of the Janus architecture, encapsulating JZ herbal medicine and FIN, respectively. A custom-designed spinneret—comprising two parallel stainless steel tubes nested within a plastic sheath—was engineered to enable side-by-side fiber formation. **Results**: Scanning and transmission electron microscopy confirmed linear morphologies with a definitive side-by-side Janus structure. X-ray diffraction and Fourier Transform Infrared Spectroscopy revealed that all active ingredients were dispersed in an amorphous state, reflecting polymer–drug compatibility. Encapsulation efficiencies reached 96.34 ± 0.47% for FIN and 94.15 ± 0.48% for JZ herbal medicine. A newly devised water-droplet assay demonstrated that almost all the nanofibers exhibited the intended side-by-side configuration, as evidenced by the rapid dissolution of the PVP side. In vitro release studies showed an initial pulsatile burst of JZ herbal medicine followed by a sustained FIN release profile, as suggested by the single-drug-loaded Janus nanofibrous controls. **Conclusions**: The present Janus nanostructure system, fabricated via a facile co-shell solvent electrospinning process, has the potential to enable the concurrent yet asynchronous delivery of FIN and JZ herbal components within a single nano-dosage form. This conceptual advance expands the toolkit for designing combination nanomedicines, allowing independent modulation of release kinetics for individual drugs to maximize prospective joint efficacy.

## 1. Introduction

Modernization of traditional Chinese medicine is a long-term development strategy of the pharmaceutical industry supported by the state [1,2,3]. The integration of traditional Chinese and Western medicine represents a viable and effective pathway for the implementation of the development strategy [4,5,6]. In practice, clinicians prescribe a variety of drugs to patients at the same time to treat a disease, which often include a small-molecule Western medicine and a multi-component Chinese herbal medicine. Due to the different pharmacology of treatment, the combination of Chinese and Western medicine can often produce a synergistic effect that allows for better therapeutic effects. However, due to the different dosages, proportions and administration methods of multiple drugs, patients may experience difficulty taking them. Provided that both traditional Chinese and Western medicine can be encapsulated into a single dosage form and simultaneously manipulated to achieve controlled release in a reasonable manner, more convenience, safety and efficiency may be offered to patients. Thus, new pharmaceutical techniques, particularly nanotechniques and their related complex architectures, are highly desired for fulfilling this goal [7,8].

Polymer has acted as a backbone to support the innovations and developments of modern drug delivery systems during the past half-century [9,10]. Accordingly, techniques for treating and converting polymer-based composites and hybrids are frequently introduced into this applied field. Among them, electrospinning is one of the most popular ones due to the advantages of the working processes (one-step, straightforward, convenient operation on an inexpensive apparatus) and also the resultant products (nanofibers or nanostructures, high surface area, random or organized fibrous films, high porosity and ease of further conversions) [11,12,13,14,15,16]. Thus, it is no surprise that electrospun nanofiber-based drug delivery systems (DDSs) have increased sharply during the past two decades. However, among those DDSs, Janus nanofiber-based DDSs are extremely rare [17], although Janus structure, as a counterpart of core–sheath structure [18,19], represents one of the most fundamental configurations for developing all kinds of functional materials.

Electrospinning was revived due to the surge of nanotechnology and is receiving more and more attention in both social and academic regions in this nano era [19,20]. Catering to the three directions of nanoscience and nanoengineering (i.e., smaller for picotechnology, complex nanostructures and nanodevices, and ordered alignment and patterning), electrospinning has been continuously developed and differentiated [21]. On one hand, depending on the target nanostructure, electrospinning can be performed via co-axial or side-by-side setups, allowing core–sheath and Janus architectures to be produced directly and in a single step [22,23,24,25,26]. Although both core–sheath and Janus nanostructures are the most fundamental bi-chamber ones, investigations about their drug delivery applications showed a world of difference. A simple search in Web of Science (3 July 2026) by topic “(core–sheath or core–shell) and electrospinning and drug delivery” and “(Janus or side-by-side) and electrospinning and drug delivery” yielded 577 and 66 results, respectively. Excluding those review articles, the ratio of 41/457 = 0.08972 is even smaller. This is abnormal. The giant difference should be attributed to their implementation difficulties, not their fundamental support for developing functional devices and systems.

On the other hand, based on the number of working fluids simultaneously delivered through a single nozzle, electrospinning can be categorized into single-fluid blending electrospinning—which accounts for over 95% of reported studies—bi-fluid co-axial and side-by-side electrospinning, as well as tri-fluid and even quad-fluid electrospinning [27,28,29,30]. Although reports about bi-fluid side-by-side fluid electrospinning are limited, bi-fluid co-axial and even tri-fluid co-axial (often abbreviated as triaxial) electrospinning have been extensively investigated [31,32,33,34,35]. This should be attributed to the fact that there is always only one working fluid contacting the environment regardless of whether the setup is co-axial, triaxial or even quad-axial [29]. This arrangement greatly simplifies the complexity of the process by eliminating the negative influences between multiple working fluids and the environments. Building on this insight, we hypothesized that a co-fluid could be incorporated into the side-by-side fluid for initiating the working process, and furthermore, the co-fluid could be a pure solvent or a solvent mixture to produce the final integrated bi-compartmental Janus nanostructure!

For “safe, efficacious and convenient” drug delivery, core–sheath nanostructures are continuously exploited to develop new strategies for tailoring components, compositions and spatial distributions [17,36]. For example, radial gradient distributions of polymers or drugs, functional coatings, blank coatings, and inner core drug reservoirs have been reported [37,38,39]. Compared with a core–sheath nanostructure, Janus nanostructures have their unique property, i.e., both sides directly contact the environment, through which material exchange and transfer can be carried out simultaneously for combined effects, synergistic actions, or multiple functional performances. Inspired by other fundamental bi-chamber nanostructures, i.e., core–sheath nanofibers and core–shell nanoparticles for co-loading two drugs, we put forward a proof-of-concept hypothesis that Janus nanofibers can be exploited to load two drugs. Furthermore, Janus nanofibers are expected to enable fast release of a Tibetan medicine from one side and extended release of a Western medicine from the other; both drugs and their release profiles can be manipulated through reasonable selection of the polymeric matrices.

To demonstrate this new concept, polyvinylpyrrolidone (PVP) and ethylcellulose (EC) were exploited as the soluble and insoluble matrices, respectively. EC was used for the sustained release of finasteride on one side of the Janus nanofibers, while PVP enabled the fast release of the Tibetan medicine from the other side. Both EC and PVP are popular drug carriers, common excipients in the pharmaceutical industry and also in the laboratory, and are mainly exploited for extending or accelerating release due to their different solubility [40,41,42,43], respectively. Finasteride is a small-molecule Western medicine. Sustained release is desired for treating prostatitis in patients [44]. As a herbal medicine for the same targeted applications but through different mechanisms, Jingzhu Tibetan capsules were selected to be encapsulated into the crescent sides of Janus nanofibers, which are a popular commercial product provided by the Jingzhu Tibetan Medicine High & New-tech Industry Co., Ltd. (Xining, China. Lot No.: GUOYAOZHUNZI Z20025304, executed standard: In kidney volume of Collection of National Standards of Chinese Medicine: WS-10251(ZD-0251)-2002-2012Z). The medicated Janus nanofibers were evaluated in terms of morphologies, inner configurations, physical state and compatibility of the components, encapsulation efficiency and in vitro dissolution tests to demonstrate the new concept for developing Janus nanofiber-based nano DDSs.

## 2. Materials and Methods

### 2.1. Materials

Jing-Zhu Qianlie Longbitong Jiaonang (a Tibetan drug, abbreviated as JZ herb medicine, Lot No.: GUOYAOZHUNZI Z20025304) was produced by Qinghai Jingzhu Tibetan Medicine High & New-tech Industry Co., Ltd., Xining, Qinghai Province. Its specification is included in Section S1 in the Supplementary Materials. Finasteride (FIN, with a purity of 98%) was bought from Meryer (Shanghai) Chemical Technology Co., Ltd. (Shanghai, China). The commercial FIN tablets were purchased for comparison from Hangzhou CONBA Pharmaceutical Co., Ltd. (Hangzhou, China). Its details are included in Section S2 in the Supplementary Materials. Polyvinylpyrrolidone (PVP, K90 grade, M_w_ = 1,300,000), ethylcellulose (EC, viscosity 6–9 mPa·s), anhydrous ethanol, N,N-dimethylacetamide (DMAc), and dichloromethane (DCM) were supplied by SinoPharm Chemical Reagent Co., Ltd. (Shanghai, China). Phosphate-buffered solution (PBS, 0.1 M, pH 7.0) was acquired from Tianjin Zhiyuan Chemical Reagent Co., Ltd. (Tianjin, China). Water was freshly double-distilled prior to each experiment. All reagents were employed as received without additional treatment.

### 2.2. Modified Electrospinning

A brand new tri-channel spinneret, composed of two parallel stainless steel capillaries nested into a Teflon tube, was developed with reference to our previous work and a Chinese patent [45,46]. With the structural spinneret as the key element, a homemade electrospinning apparatus was set up for implementing the tri-fluid side-by-side electrospinning. The remaining components include the following: (1) three syringe pumps (KDS 100 and KDS 200, Cole-Parmer, Vernon Hills, IL, USA) for precisely delivering the three working fluids to the spinneret at controlled flow rates; (2) a high-voltage power supply (model 2000 ZGF, 60 kV/2 mA, Wuhan Huatian High Power Co., Ltd., Wuhan, China); and (3) a fiber collector constructed from aluminum foil-wrapped cardboard. Other auxiliary devices include a glare lamp (MJSDT001QW, Shanghai Qiwai Technology and Culture Co., Ltd., Shanghai, China), a smartphone (Huawei Pro60, Shenzhen, China), and a camera (Canon G7X, Tokyo, Japan), which were utilized to investigate the working processes.

Several working fluids were prepared for preparing five kinds of nanofibers, whose electrospinning conditions are included in Table 1. Based on a previous reported method [24], nanofibers F3-JZ and F3-FIN were prepared for evaluating the drug encapsulation efficiencies and in vitro dissolution profiles of JZ herbal medicine and FIN in the Janus nanofibers F3.

### 2.3. Characterizations

#### 2.3.1. Morphologies

The morphologies of the prepared nanofibers including the monolithic nanofibers F1, F2 and the Janus nanofibers F3 were evaluated using a scanning electron microscope (Quanta FEG450, FEI Corporation, USA). Optical microscopic images of all the prepared nanofibers are included in Section S3 of the Supplementary Materials. A sheet of the nanofibrous mat was cut and attached on the conductive adhesive. The fibrous mats were coated with Pt for over 60 s under a nitrogen ambient. The applied voltage and the magnification were fixed at 20 keV and 20,000, respectively. The nanofibers‘ diameters were estimated through ImageJ software V1.8.0 (National Institutes of Health, Bethesda, MD, USA) on about one hundred places on the SEM images.

#### 2.3.2. Inner Side-by-Side Structures

The inner structures of nanofibers F1, F2 and F3 were assessed using a transmission electron microscope (TEM, JEM2200F, JEOL, Japan). TEM samples were prepared by placing a carbon film supported by 200-mesh copper mesh on the collector for about 10 s during the electrospinning process, followed by placing the collected nanofibers into the vacuum chamber for evaluation. The applied accelerated voltage for evaluation was 80 kV.

#### 2.3.3. Physical State

The physical state of raw PVP, EC, JZ herbal medicine, and FIN powers and their electrospun composite nanofibers were measured through an X-ray diffractometer (XRD, Bruker-AXS, Karlsruhe, Germany) using a graphite monochromator for radiation of Cu-Kα. The radiation source was run under an applied voltage of 45 kV and a current of 40 mA. The 2θ scanning range was from 5° to 60° with a fixed speed of 10°/min.

#### 2.3.4. Compatibility Among the Components in the Janus Nanofibers

The Attenuated Total Reflectance–Fourier Transform Infrared Spectroscopy (ATR-FTIR) spectra of the four raw particles (JZ herbal medicine, FIN, PVP and EC) and their electrospun nanofibers were determined through a Spectrum 100 Fourier Transform Infrared Spectroscopy (Billerica, MA, USA). All the ATR-FTIR spectra were achieved under ambient conditions. The scanning range was from 500 cm^−1^ to 4000 cm^−1^ with a resolution of 2 cm^−1^. The scanning time was set to 8 times for each sample.

### 2.4. Functional Performance Evaluations

#### 2.4.1. Drug Encapsulation Efficiency

A series of standard solutions of JZ herbal medicine and FIN were prepared and were scanned through a UV-2102PC Spectrophotometer (Unico Instrument Co., Ltd., Shanghai, China). Their maximum absorbances at 220 nm were exploited to quantitatively measure FIN contents in its solutions. JZ herbal medicine is a mixture of multiple components. However, its solution has a maximum absorbance at 281 nm. This suggests that there are one or more components that contain benzene rings in their molecules, and these components can be exploited as representatives to evaluate the drug encapsulation efficiency (DEE%) of JZ herbal medicine in nanofibers F3-JZ. In other words, the quantification of the JZ formulation was a JZ-equivalent determination rather than a specific quantification of all JZ components.

DEE% is an important parameter for evaluating the pharmaceutical nanotechniques and their resultant nano DDSs [38,47]. It can be measured according to the following procedures: An amount of the electrospun nanofibrous mats F1, F2, and the one-drug controls of Janus nanofibers F3 (i.e., F3-JZ and F3-FIN) was accurately weighted. The mats were dissolved in 10 mL anhydrous ethanol. The solutions were dripped into 100 mL phosphate-buffered solution (PBS, pH 7.0, 0.1 M) under magnetic stirring. One milliliter was drawn, reasonably diluted using PBS and passed through a 0.22 µm MCE membrane (Navigator Lab Instrument Co., Ltd., Tianjin, China) prior to UV measurements. Based on the predetermined calibration equation, the concentrations could be calculated. Thereafter, the value of DEE% could be calculated through Equation (1):

(1) DEE(%)=WdWp×100%

where DEE(%) is the encapsulation efficiency; W_d_ is JZ herbal medicine or FIN determined in the electrospun nanofibers; and W_p_ represents the JZ herbal medicine or FIN contents theoretically added in the working fluids during the preparation of nanofibers. All measurements were carried out with three small different patches cut from the collected electrospun nanofibrous films.

#### 2.4.2. Optical Microscopic Observation of the Asynchronous Drug Delivery

To achieve intuitive and direct observation of the asynchronous drug delivery, an optical microscope (SK2009HDMI-TH3, Shenzhen Saike Digital Technology Development Co., Ltd., Shenzhen, China) was used to observe the dissolution process of the collected Janus nanofibers placed on a glass slide under ambient conditions at a magnification of 200×. A water droplet of approximately 3 µL was dispensed onto the nanofibers using a syringe. S-EYE software (Shenzhen Saike Digital Technology Development Co., Ltd., Shenzhen, China) was used to record the entire dissolution process for image acquisition and processing.

#### 2.4.3. In Vitro Dissolution Tests

An SHZ-86 constant-temperature water-bath shaker (Changzhou Jintan Shuibeike Pu Experimental Instrument Factory, Changzhou, China) was used for the in vitro dissolution test. The conditions were fixed at 50 rpm and 37 ± 1 °C. A certain amount of nanofibers—F1, F2, F3-JZ, F3-FIN—commercial FIN tablets and JZ herbal medicine capsules were placed into six vessels. The vessels contained 600 mL PBS. The fast-release JZ herbal medicine was sampled up to 7 times within 2 h. The sustained-release FIN was sampled up to 8 times within 36 h. The withdrawn 5.0 mL aqueous solutions were filtered through a 0.22 µm MCE membrane (Navigator Lab Instrument Co., Ltd., Tianjin, China) and diluted by an appropriate factor prior to UV measurement. The filtrates were measured for their maximum absorbances. The vessels were compensated with 5.0 mL fresh PBS. All the experiments were conducted in triplicate. The in vitro dissolution profiles of JZ herbal medicine from F3-JZ and FIN from F3-FIN were exploited to jointly evaluate the asynchronous delivery of JZ herbal medicine and FIN from the Janus nanofibers F3 as a previous report [24].

### 2.5. Statistical Analysis

All experiments were performed with at least three different patches cut from the collected nanofibrous membranes, and the experimental data were expressed as mean ± standard deviation. Statistical significance was assessed using one-way analysis of variance (ANOVA) with Tukey’s test for multiple comparisons (GraphPad Prism software version 6.0). *p* < 0.05 was considered to indicate a statistically significant difference between groups.

## 3. Results

### 3.1. Modified Tri-Fluid Electrospinning Processes for Creating Janus Nanostructures

**Figure 1:**
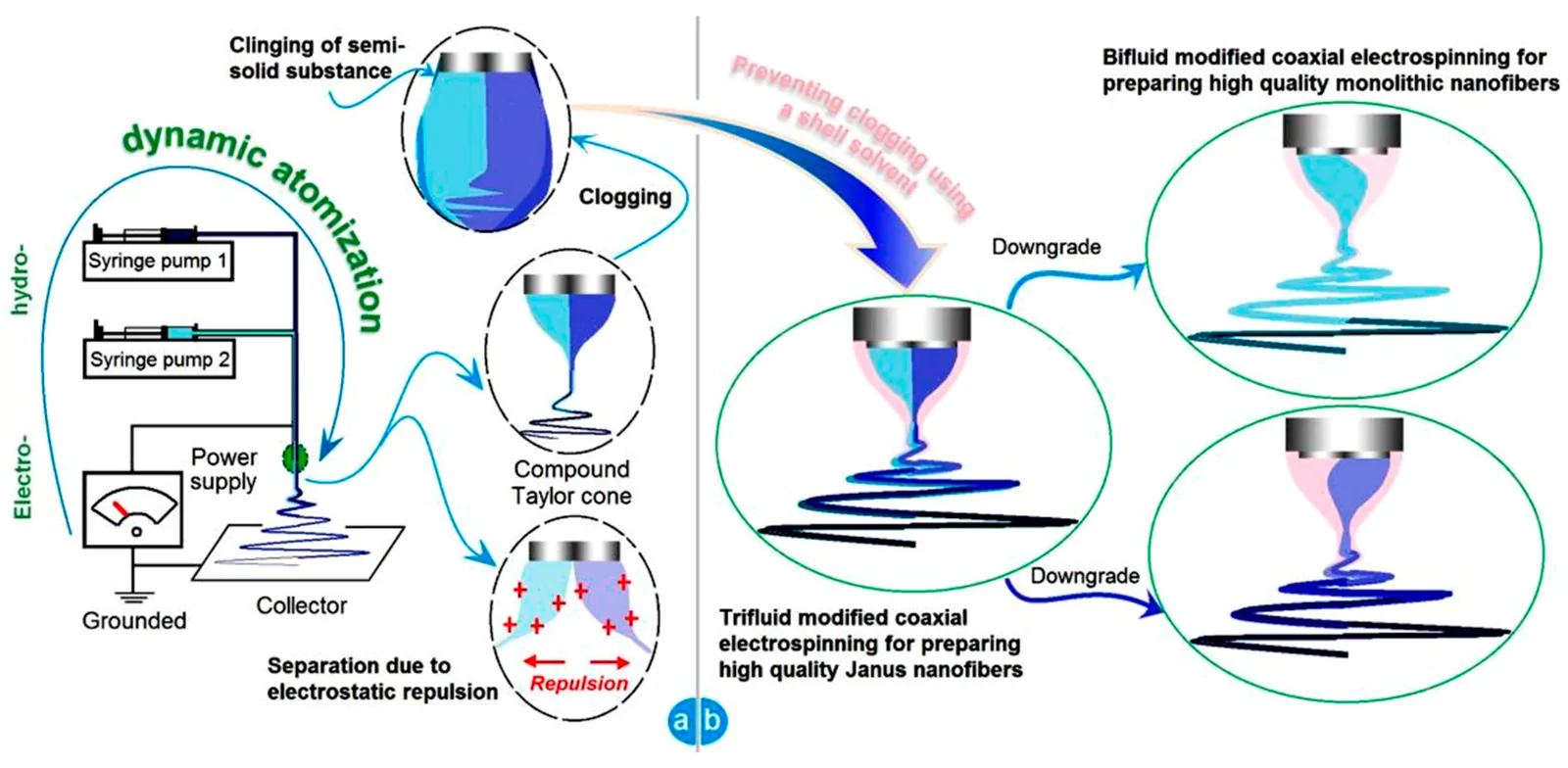
The apparatus of side-by-side electrospinning and the abnormal phenomena during traditional side-by-side electrospinning processes (**a**); the three kinds of modified co-axial electrospinning processes based on a tri-channel spinneret (**b**).

As shown in the schematic in Figure 1a, an electrospinning apparatus is mainly composed of four parts, similar to other electrohydrodynamic atomization methods: a power supply for generating the “electro-” component, two syringe pumps for the “hydro-” component, a spinneret for guiding the working fluids into the electric field in an organized manner and for the dynamic atomization of “electro-” and “hydro-”, and a collector for the deposition of the resultant nanofibers.

It is easy to think that a spinneret with two parallel metal capillaries was utilized to conduct the side-by-side electrospinning for creating Janus nanofibers, and this approach has been demonstrated previously [48,49]. However, as shown in the bottom-down inset of Figure 1a, the two working fluids are easy to separate from each other because they carry like charges and make contact at a single point when they are pumped from the nozzle. The electric repulsion is inevitable. Thus all the previous researchers had to use working fluids that have a high viscosity to provide a large adhesive force to bind each other to resist the electric repulsion [50,51]. However, another issue arises, high viscosity means a large polymeric concentration, by which the spinneret is prone to clogging, and a compound Taylor cone is rarely formed, as diagrammed in the middle-right inset of Figure 1a. Particularly, some polymers with natural sources are more prone to adhering to the metal spinneret, forming a semi-solid substance on the surface of fluid jets, distorting the working process or even completely halting the production of nanofibers [52,53].

To avoid the spinneret clogging for a continuous and robust electrospinning process, the intermittent manual addition of organic solvent has been reported to lubricate the process [54]. Meanwhile, modified co-axial and even triaxial electrospinning processes using an outer organic solvent have been reported. Thus, here, an additional third fluid, i.e., a co-shell solvent or solvent mixture of both sides, was exploited to ensure a robust side-by-side electrospinning for achieving high-quality Janus nanofibers (Figure 1b). Furthermore, when one of the two side fluids was stopped, the tri-fluid electrospinning process was downgraded into a typical bi-fluid-modified co-axial electrospinning process, which can be utilized for preparing high-quality monolithic nanofibers, as indicated by the two right insets in Figure 1b.

**Figure 2:**
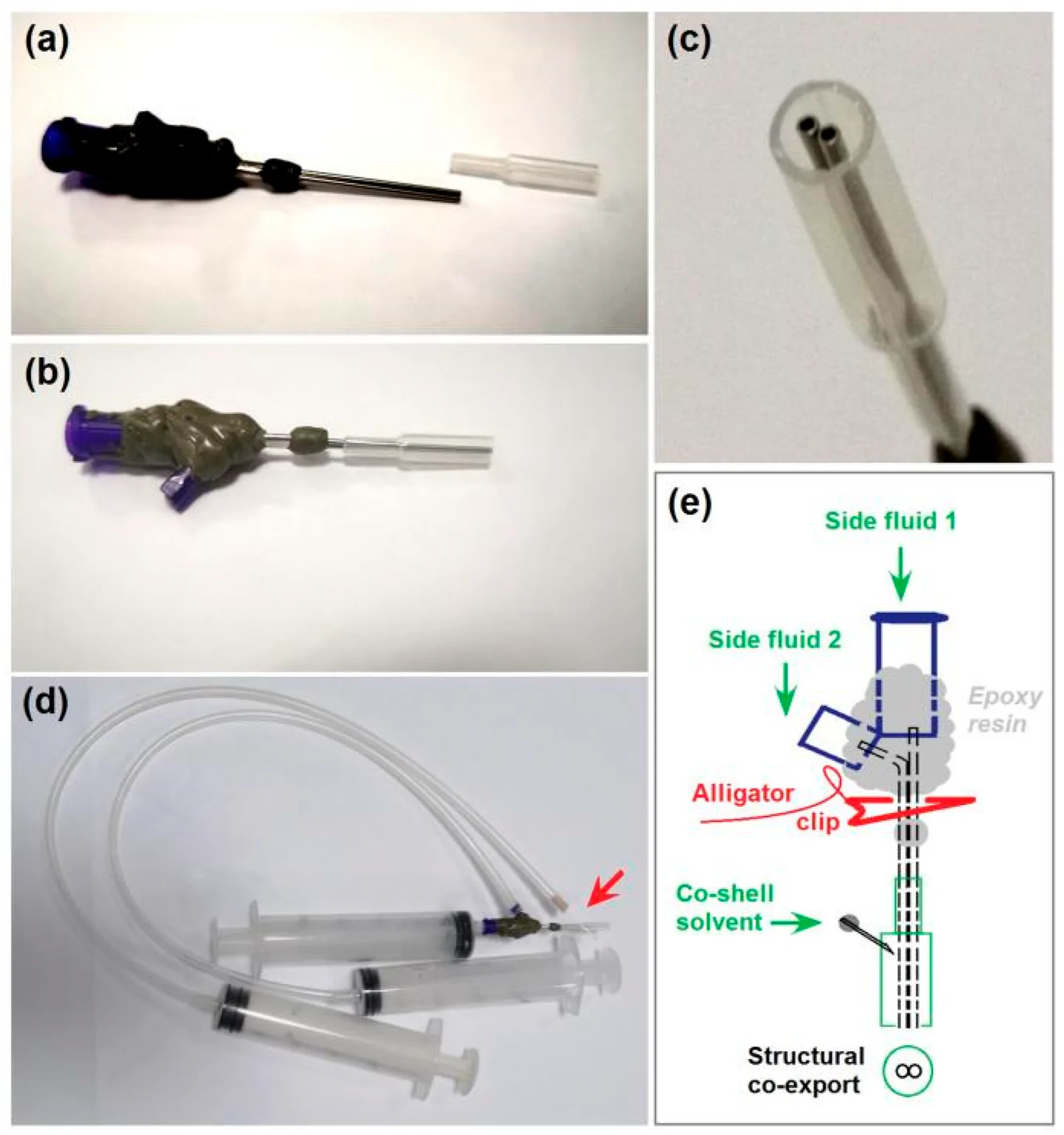
The specially designed structural spinneret: (**a**) a conventional side-by-side spinneret comprising two parallel metal capillaries and a PP tube section with variable inner diameters; (**b**) the co-axial fluid-guiding outlet formed by simply inserting the PP tube over the metal capillaries; (**c**) a digital photograph of the tri-fluid co-existence nozzle of the structural spinneret; (**d**) the spinneret connected to three syringes loaded with three distinct working fluids, where a sharp needle is inserted through the PP tube to directly deliver the outer co-axial fluid, as indicated by the red arrow; (**e**) a schematic illustration revealing the internal structural characteristics of the spinneret.

Previously, it has been reported that two viscous side-by-side fluids were covered by a common solvent for creating Janus nanofibers [45]. A whole metal tri-channel spinneret with two parallel capillaries nested in a large capillary was exploited to implement the tri-fluid side-by-side electrospinning process. Here, a pick and mix spinneret was developed for the materials conversions, which is more reasonable and straightforward than the previous one. The new spinneret comprising two parallel metal capillaries was nested into a polypropylene (PP) plastic pipe with large and small heads. The preparation of the spinneret is shown in Figure 2. Firstly, a traditional side-by-side spinneret composed of two parallel metal capillaries and a PP tube with two sections having various diameters is prepared (Figure 2a). Later, a simple insertion of the PP tube on the metal capillaries with the smaller diameter section can form the tri-fluid exit, in which the parallel metal capillaries have the common outer sheath (Figure 2b). A digital image of the tri-fluid co-existence nozzle of the structural spinneret is shown in Figure 2c. The connections of the structural spinneret to three syringes containing the three different working fluids are shown in Figure 2d. An additional sharp needle is exploited to penetrate the PP tube to directly guide the outer co-shell fluid as indicated by the red arrow. Except a section of metal parallel capillaries for connecting the alligator clip to transfer the high voltage electrostatic energy to the working fluids, other sections of the structural spinneret were covered by isolated materials, including PP tube and adhesive epoxy resin, as indicated in the diagram in Figure 2e. Based on this new concept idea of organizing spinneret with an integration of metal capillaries and isolated polymeric tubes, many other structural spinnerets can be similarly developed for creating nanofibers with hierarchical architectures.

### 3.2. Implementations of the Three Types of Modified Co-Axial Electrospinning

**Figure 3:**
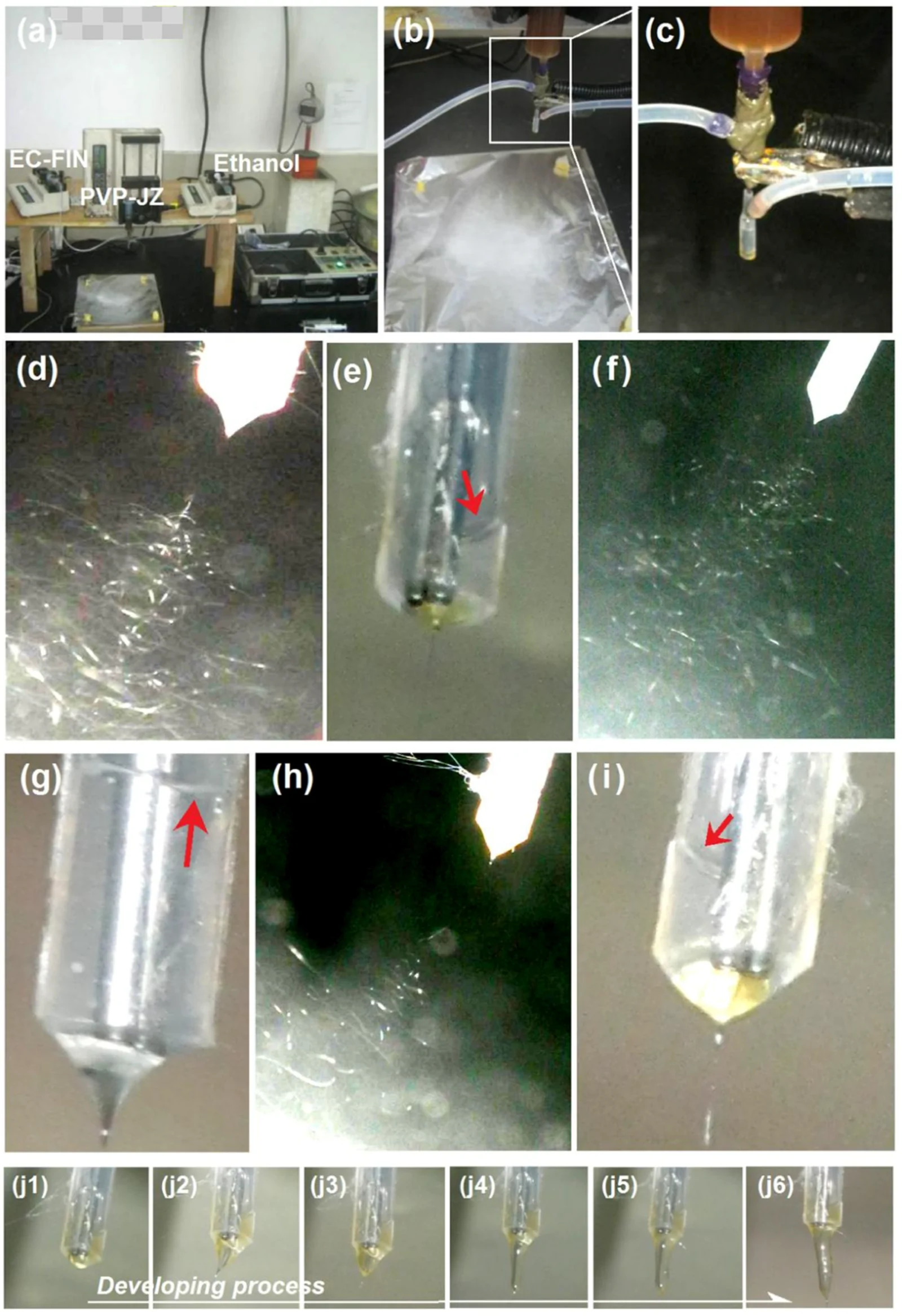
The implementations of the series of the modified co-axial electrospinning processes: (**a**) a bird’s-eye view of the homemade electrospinning apparatus; (**b**) the electro- and hydro-connection of the spinneret; (**c**) an enlarged image of the connections; (**d**) a typical electrospinning process for the preparation of nanofibers F1 loaded with PVP and JZ herbal medicine; (**e**) a compound Taylor cone and the liquid level in the PP tube for creating F1, as indicated by the red arrow; (**f**) a typical electrospinning process for the preparation of nanofibers F2 loaded with EC and FIN; (**g**) a compound Taylor cone and the liquid level in the PP tube for producing F2, as indicated by the red arrow; (**h**) a typical electrospinning process for the preparation of the Janus nanofibers F3; (**i**) a compound tri-fluid Taylor cone and the liquid level in the PP tube for producing F3, as indicated by the red arrow; (**j1**–**j6**) the gradual development of the clogging of the spinneret by the semi-solid substance when the co-shell solvent was stopped for preparing Janus nanofibers F3, the whole clogging process took about three mins.

The implementations of the three modified co-axial electrospinning processes are included in Figure 3. A bird’s-eye view of the homemade electrospinning apparatus is shown in Figure 3a. Three pumps were utilized to quantitatively drive the three working fluids to the spinneret. A power supply was exploited to provide the applied voltage. A cardboard wrapped with an aluminum foil for the deposition of electrospun nanofibers. The syringe containing PVP-JZ solution was directly inserted into the structural spinneret to fix it. The other two fluids of co-shell ethanol and EC-FIN solution were transferred to the spinneret through two silicon tubes.

The image of the convergent places around the spinneret is shown in Figure 3b and the further enlarged image is shown in Figure 3c. A typical electrospinning process of the side fluid of PVP and JZ herbal medicine for preparing the nanofibers F1 is shown in Figure 3d. The three working steps, i.e., Taylor cone, straight fluid jet, and the unstable bending and whipping region, are obvious. An enlarged image of the compound Taylor cone (formed by a surrounding of PVP-JZ fluid by the co-shell ethanol) and the liquid of ethanol level in the PP tube (as indicated by the red arrow) for generating the nanofibers F1 is shown in Figure 3e. When the PVP-JZ fluid was closed and the FIN-EC fluid was switched on, the apparatus was conducted to prepare the nanofibers F2. A typical working process is shown in Figure 3f. An enlarged image of the Taylor cone is exhibited in Figure 3g. The surface of the co-shell ethanol was clear, as indicated by the red arrow. When both side fluids were switched on, the electrospinning was implemented for creating the Janus nanofibers F3 (Figure 3h). It is obvious that the straight fluid jet is longer than those for preparing nanofibers F1 and F2. The compound Taylor cone composed of the three working fluids in Figure 3i showed an obviously larger volume than those two fluid Taylor cones. Still, the level of the co-shell ethanol can be discerned, as indicated by the red arrow. When the co-shell ethanol was closed, a typical process gradual development of the clogging of the spinneret by the semi-solid substance was recorded from Figure 3(j1–j6). The whole clogging process took about three mins.

### 3.3. Morphologies and Inner Structures of the Produced Nanofibers

**Figure 4:**
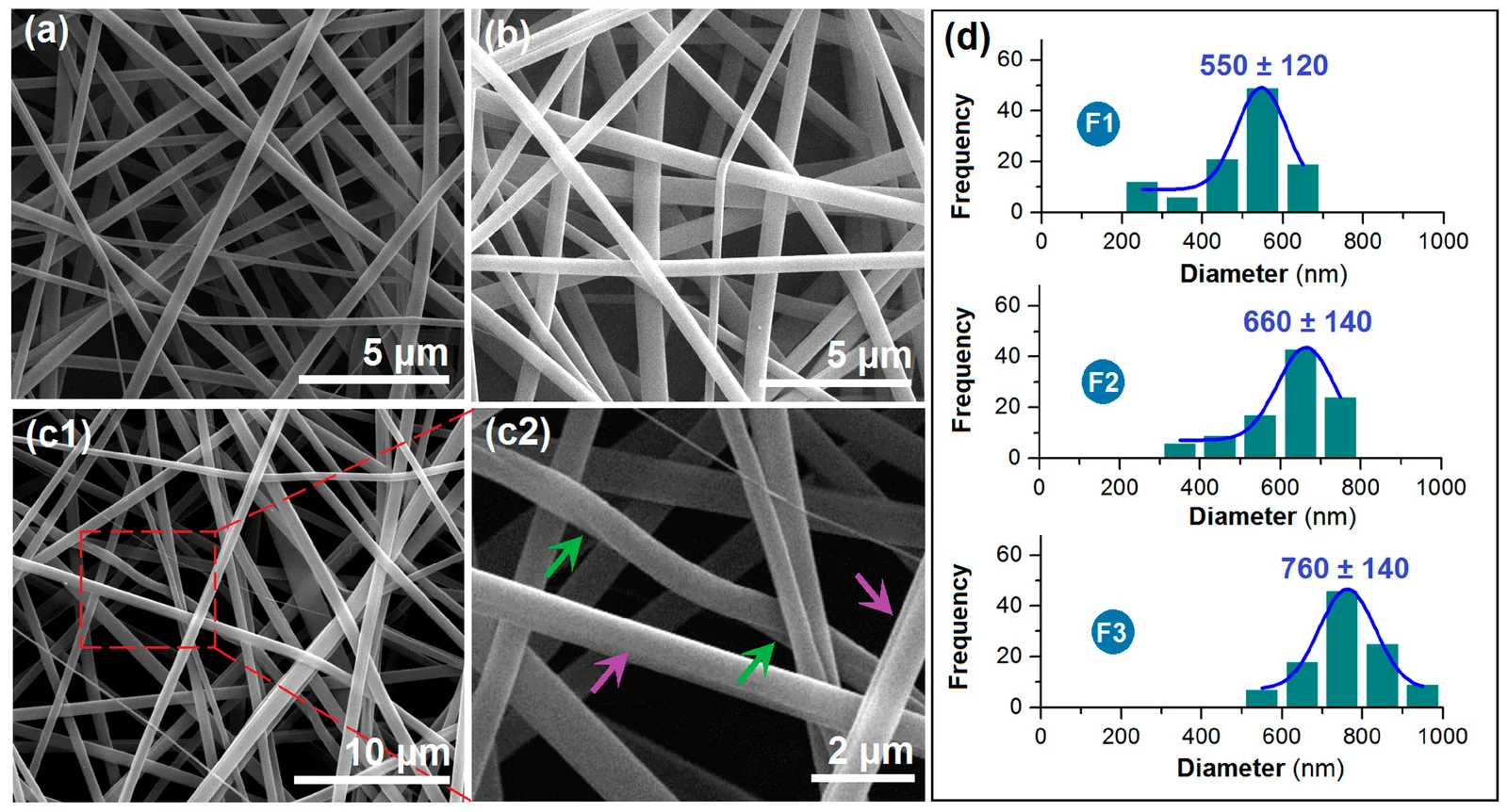
SEM images of the three types of electrospun medicated nanofibers: (**a**) nanofibers F1; (**b**) nanofibers F2; (**c1**,**c2**) Janus nanofibers F3; and (**d**) diameter and diameter distributions of nanofibers F1 to F3.

The SEM images of the three kinds of electrospun medicated nanofibers are included in Figure 4. It is clear that all the three kinds of nanofibers have linear morphologies. No round particles or elliptical spindles are found among the nanofibers F1 (Figure 4a) and F2 (Figure 4b). These phenomena suggest that both the PVP-JZ and EC-FIN have good electrospinnability. When these two fluids were organized in a parallel manner with a surrounding solvent mixture to experience the side-by-side electrospinning treatment, the resulting Janus nanofibers F3 mainly presented in a straight linear manner (Figure 4(c1)). However, curved nanofibers were occasionally found within the nanofibrous mats, as indicated by the green arrows in Figure 4(c2). Regardless of the curved nanofibers or the straight linear nanofibers, an additional line along the axial direction can be found on the smooth surface of the nanofibers, as indicated by the pink and green arrows in Figure 4(c2), which give a hint of the inner side-by-side structure. All three kinds of nanofibers—F1, F2, and F3—exhibited uniform diameter distributions. As shown in Figure 4d, the homogeneous nanofibersF1 and F2 had average diameters of 550 ± 120 nm and 660 ± 140 nm, respectively. In contrast, the Janus nanofibers F3 had an average diameter of 760 ± 140 nm, slightly larger than those of the homogeneous nanofibers F1 and F2.

**Figure 5:**
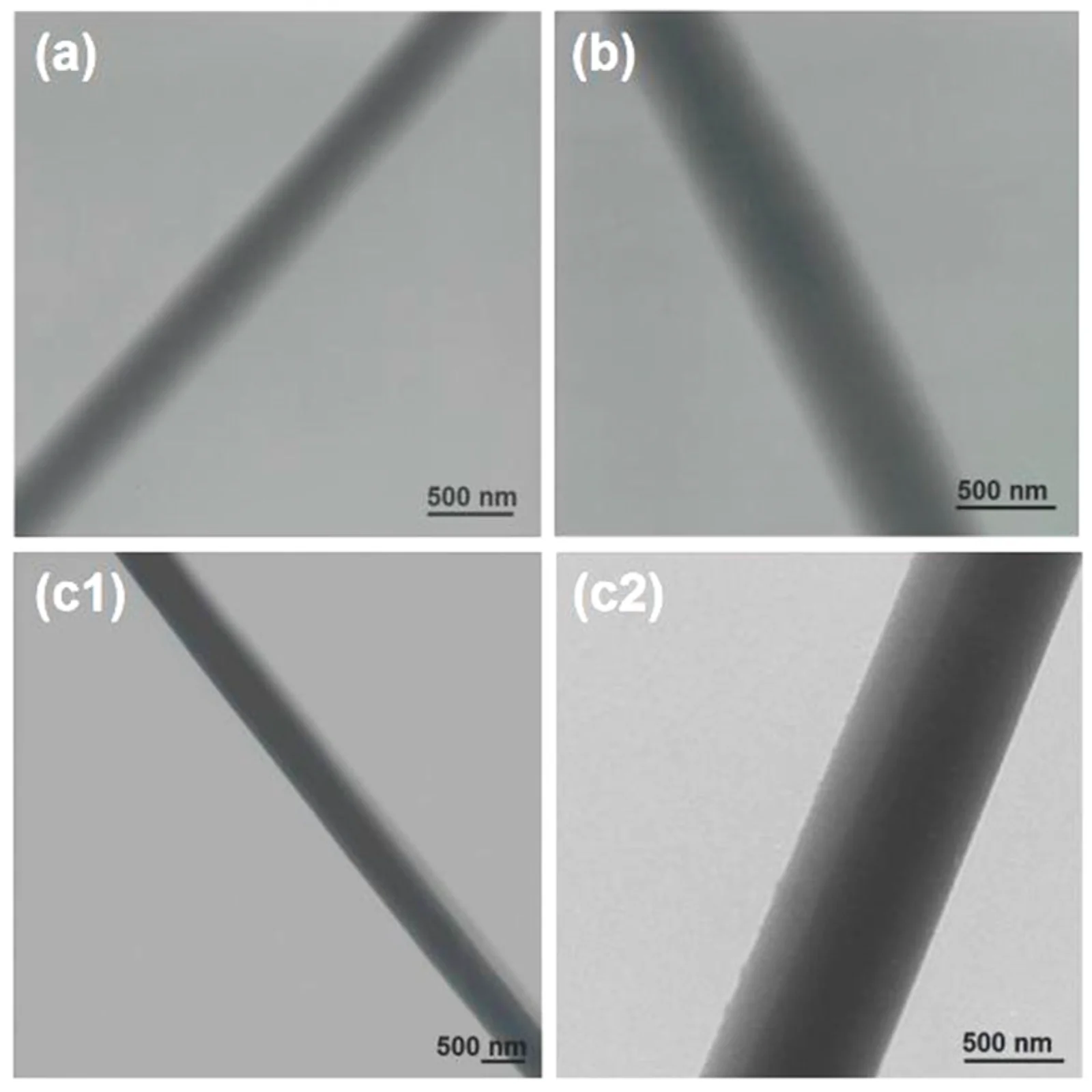
TEM images of the three kinds of electrospun medicated nanofibers: (**a**) nanofibers F1; (**b**) nanofibers F2; and (**c1**,**c2**) Janus nanofibers F3.

The inner structures of nanofibers F1, F2 and F3 were evaluated using TEM, whose images are shown in Figure 5. Just as numerous reports that the monolithic nanofibers from the single-fluid blended electrospinning, both nanofibers F1 and F2 (Figure 5a and Figure 5b, respectively) showed a gradual gray level transformation from the core to the boundary in a continuous manner. This should be attributed to the fact that the components and compositions were the same all over the nanofibers, but the thicknesses from the center to the boundary reduced gradually and continuously. However, the Janus nanofibers F3 had two distinct regions of gray levels, as indicated by the two nanofibers’ images in Figure 5(c1,c2). Although the two side fluids had the same flow rate for preparing the Janus nanofibers, they had a big difference in the concentration of the solutes. Thus, it is not strange that the EC-FIN sides had a larger gray level and a bigger thickness than those of the JZ-PVP sides within the Janus nanostructures.

**Figure 6:**
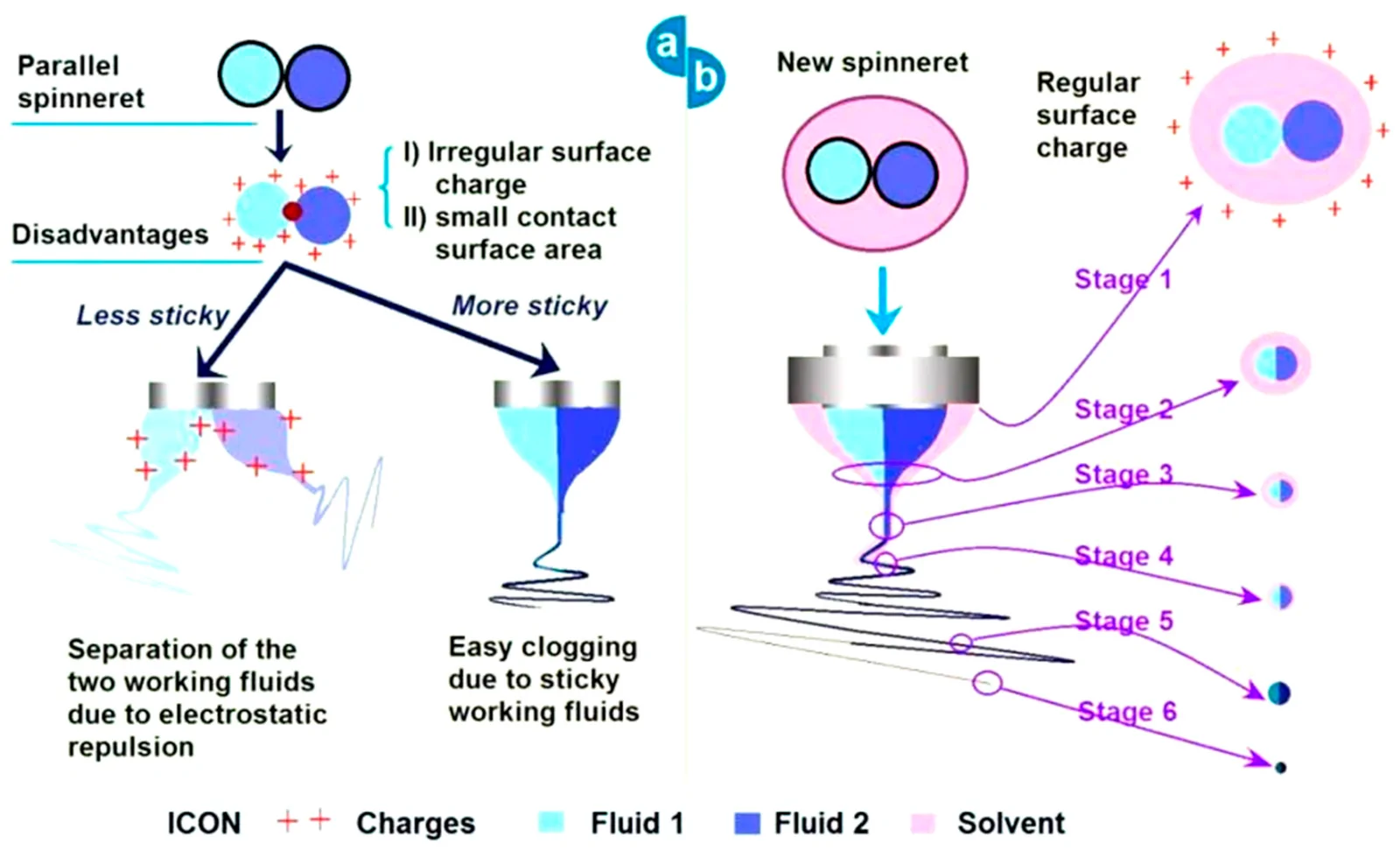
The suggested mechanisms of the influences of outer co-shell solvents on the nano fabrications of electrospun Janus nanofibers.

A diagram about the working processes and mechanisms of the traditional side-by-side electrospinning and the present modified tri-fluid electrospinning is included in Figure 6. Based on the common idea for co-axial electrospinning, i.e., core–sheath nanofibers can be directly prepared using a concentric spinneret, it is easy to take for granted that Janus nanofibers can be similarly created using a spinneret with two parallel metal capillaries (Figure 6a). However, the following three facts make the simple production of the integrated Janus nanofibers using the parallel spinneret very difficult: (1) the two fluids are firstly charged and pumped to the nozzle of the spinneret to meet each other, and later begin the electrospinning process from a side-by-side Taylor cone; (2) the nozzle spinneret comprises an irregular surface charge area or boundaries; and (3) the two fluids have a very small contact point (as indicated by the red dot in Figure 6a). These facts co-act to create a major challenge for implementing the side-by-side electrospinning, i.e., on one hand, less sticky working fluids make the two fluids easy to separate from each other even from the formation of Taylor cone besides the electrospinnability concern due to the time procedure of first charge and later meeting. Certainly, the later straight fluid jet and unstable region may also result in separation. On the other hand, a more sticky working fluid is more prone to resulting in the clogging of the spinneret.

When a co-shell solvent is applied around the two side-by-side electrospinnable working fluids, they would experience the following six stages as indicated in Figure 6b. In stage 1, the co-shell solvent not only provides a round circle for charging, but also lubricates the spinneret and facilitates the formation of a compound Taylor cone. In stage 2, the shell solvent is able to effectively cover the two side-by-side polymeric working fluids. This encapsulation can extend to the straight fluid jet section (Stage 3) and also the former period of the unstable region (Stage 4). Later, at the intermediate or last section of the bending and whipping fluids (Stage 5), the shell solvent would be exhausted, the two side working fluids become very sticky and are strong enough to prevent the separation of two fluids. Finally, the solid Janus nanofibers are formed (Stage 6) and deposited on the collector to randomly form the nanofibrous films.

Certainly, the positive influences of a co-shell solvent not only offer a round charge circle, provide a first meeting of two side-by-side fluids, later co-entering the electrical field procedure, and prevent the possible separations from the Taylor cone to the unstable region, but also ensure that the two side-by-side working fluids receive less interference from the environment, and in turn produce nanofibers with smaller diameters and more uniform diameter distributions.

### 3.4. Physical State and Compatibility

**Figure 7:**
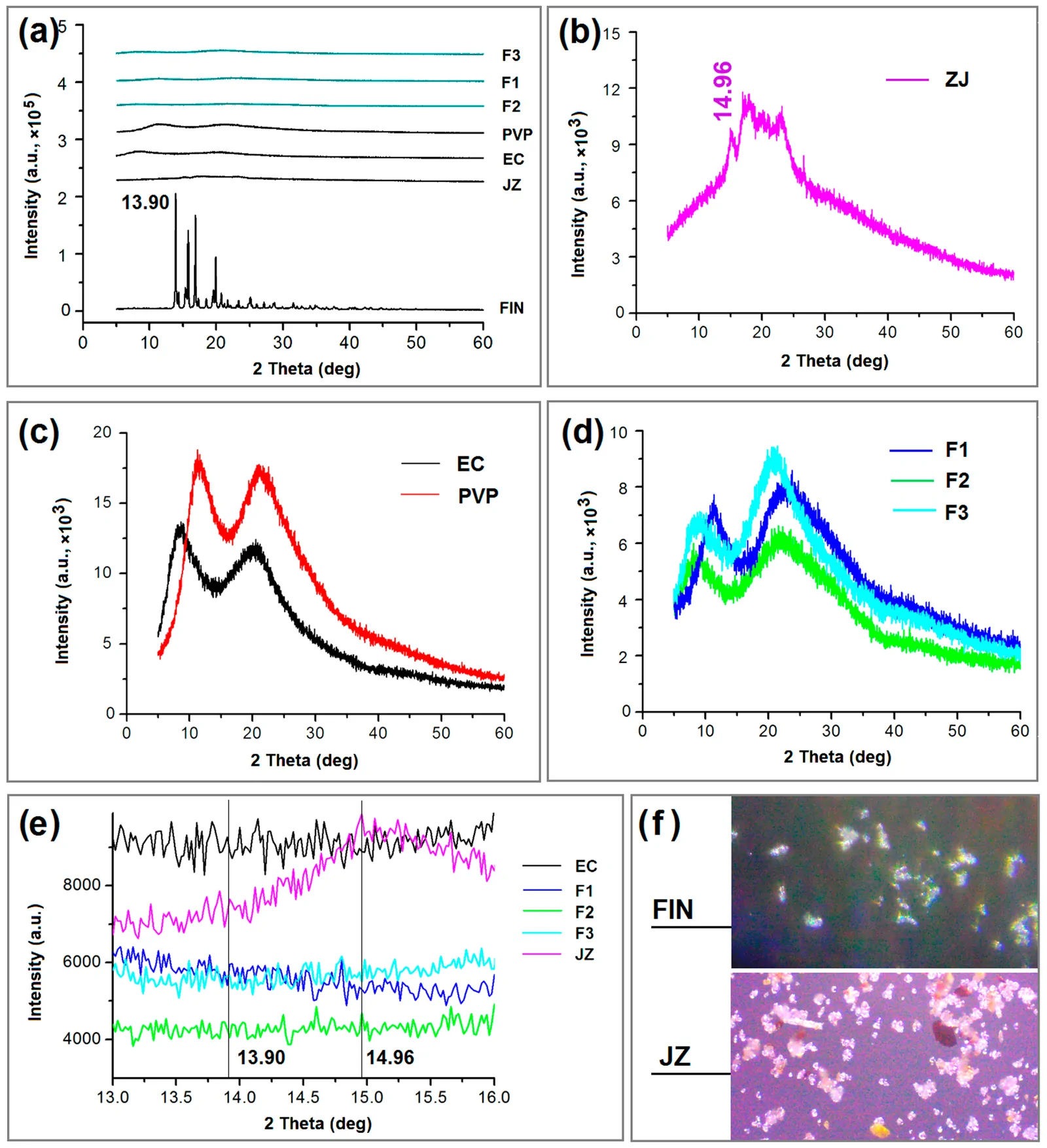
XRD patterns of the created medicated nanofibers and the powders of the raw materials (FIN, ZJ herbal medicine, PVP and EC): (**a**) the same y-axis scale range of all the tested samples; (**b**) a reduced y-axis scale range to enlarge the signals of JZ herbal medicine; (**c**) a reduced y-axis scale range to enlarge the signals of raw EC and PVP powders; (**d**) a reduced y-axis scale range to enlarge the signals of the electrospun nanofibers F1, F2 and F3; (**e**) a reduced both y and x scale range to disclose the statue of the sharp peak of 13.90 degrees of the FIN patterns and 14.96 degrees of the JZ patterns in the electrospun nanofibers F1 to F3; and (**f**) the optical microscopic images of the raw FIN and JZ herbal medicine powders.

Amorphous state is a favorable physical state for drug delivery, particularly for the large number of poorly water-soluble drugs [55,56]. The XRD patterns of the four powders of FIN, ZJ herbal medicine, PVP and EC and their electrospun medicated nanofibers are included in Figure 7. Based on the same y-scale, all the patterns are shown in Figure 7a together. For XRD patterns after 45 degrees of 2Ɵ, the patterns often have no significant scientific meanings [57,58]. XRD is mainly a qualitative method. It becomes unreliable when the content of crystalline is lower than 5% [59,60,61,62]. Thus, except for the patterns of FIN, other patterns are signified by several orders of magnitude under a smaller y-axis, their remarkable differences among these materials are obvious. The powders of JZ herbal medicine have several sharp peaks, although their intensity is not strong, as indicated by the peak of 14.96 degrees in Figure 7b. The patterns of EC, PVP (Figure 7c), and their nanofibers F1 to F3 (Figure 7d) have the typical two halos, reflecting the mainly amorphous physical state. The further reductions in the y-axis and x-axis of the XRD patterns in Figure 7e indicate the characteristic peaks of 13.90 degrees for FIN and 14.96 degrees for JZ herbal medicine completely disappeared from the patterns of their nanofibers F1, F2 and F3. These disappearances clearly demonstrated that the electrospun nanofibers are polymeric nanocomposites, and that electrospinning is a useful tool for creating amorphous solid dispersions of poorly water-soluble drugs. The crystalline properties of FIN and JZ herbal medicine can be simply observed using the optical microscope, whose images are shown in Figure 7f.

**Figure 8:**
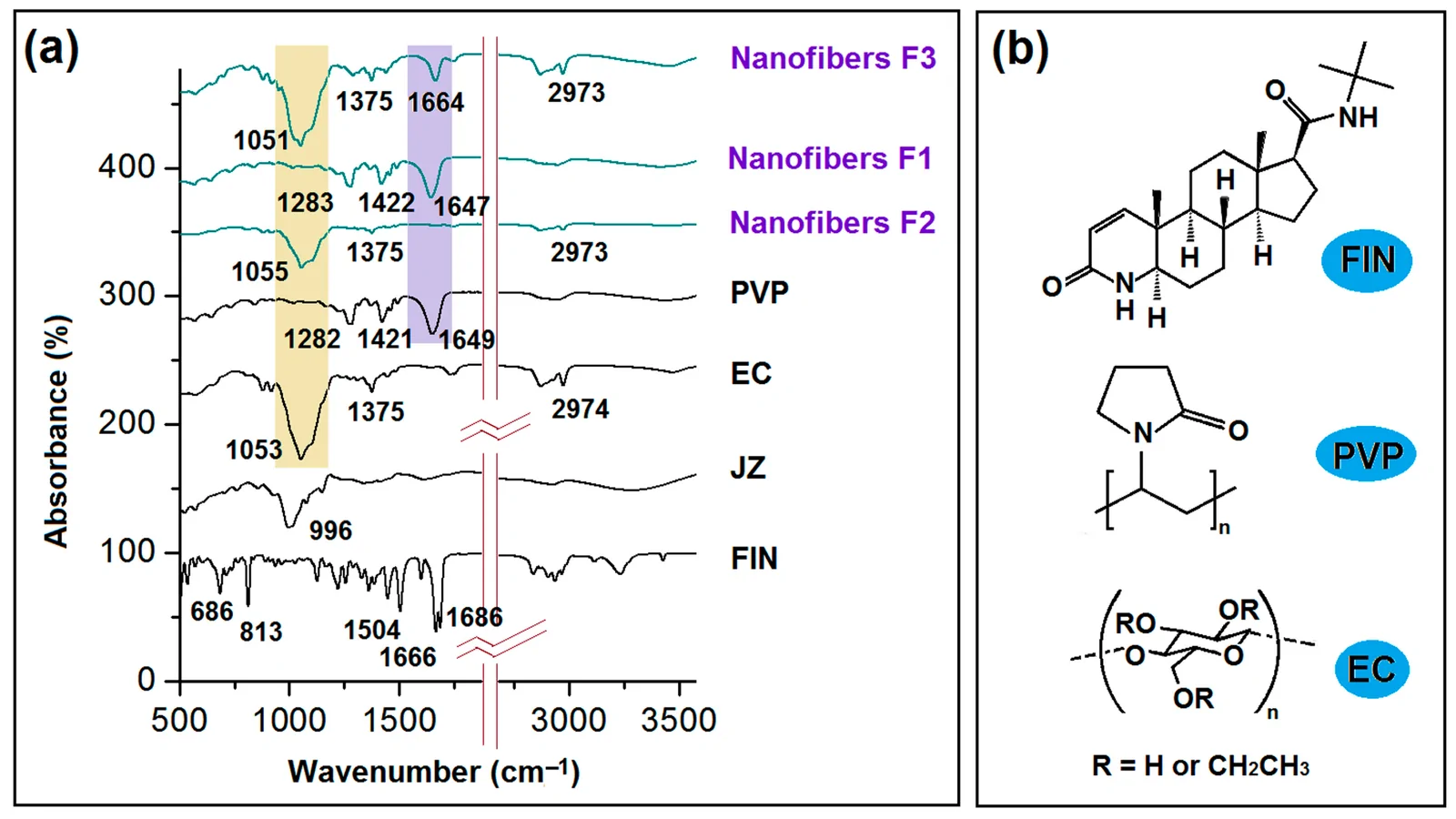
ATR-FTIR spectra of the created medicated nanofibers and the powders of the raw materials (FIN, ZJ herbal medicine, PVP and EC) (**a**) and the chemical structures of FIN, PVP and EC molecules (**b**).

The FTIR spectra of the raw powder materials (JZ herbal medicine, FIN, EC and PVP) and their created medicated nanofibers are included in Figure 8a. The chemical structures of FIN, PVP and EC molecules are shown in Figure 8b. As indicated by the light yellow region, nanofibers F3 and nanofibers F2 have the sharp peak of EC, indicating these nanofibers are EC-based nanocomposites. In contrast, the light blue region of nanofibers F3 and nanofibers F1 has the similar sharp peak of PVP, hinting that these nanofibers are PVP-based nanocomposites. The spectra of nanofibers F3, to a certain extent, are an overlapped line of the spectra of monolithic nanofibers F1 and nanofibers F2, suggesting the co-presence of both polymeric matrices EC and PVP in the nanofibers F3.

The JZ herbal medicine powders have only one sharp peak at 996 cm^−1^ in their spectra. In contrast, the FIN raw powders have a series of sharp peaks on their spectra. However, these peaks disappeared from the spectra of all three electrospun nanofibers. These phenomena should be attributed to the favorable secondary interactions between the JZ herbal medicine components and FIN with their polymeric matrices, i.e., PVP and EC, respectively. FIN molecules have C=O groups, which support the formation of hydrogen bonds with the OH groups of EC molecules besides hydrophobic and electrostatic interactions among their functional groups. Although JZ herbal medicine has multiple components, PVP molecules are able to form secondary interactions due to the C=O groups, the positively charged N atoms, and the hydrophobic carbon chains. Both PVP and EC are the most common pharmaceutical excipients broadly utilized in the pharmaceutical industry and also in experimental laboratories. The FTIR results, as anticipated, verified that the amorphous status of the double drugs when they were encapsulated within the amorphous polymeric matrices. The chemical stability, physical stability, and physiological stability during use of the medicated Janus nanofibers deserve further research.

### 3.5. Quantitative Measurements of JZ Herbal Medicine and FIN and Their Controlled Release Profiles from the Electrospun Nanofibers

**Figure 9:**
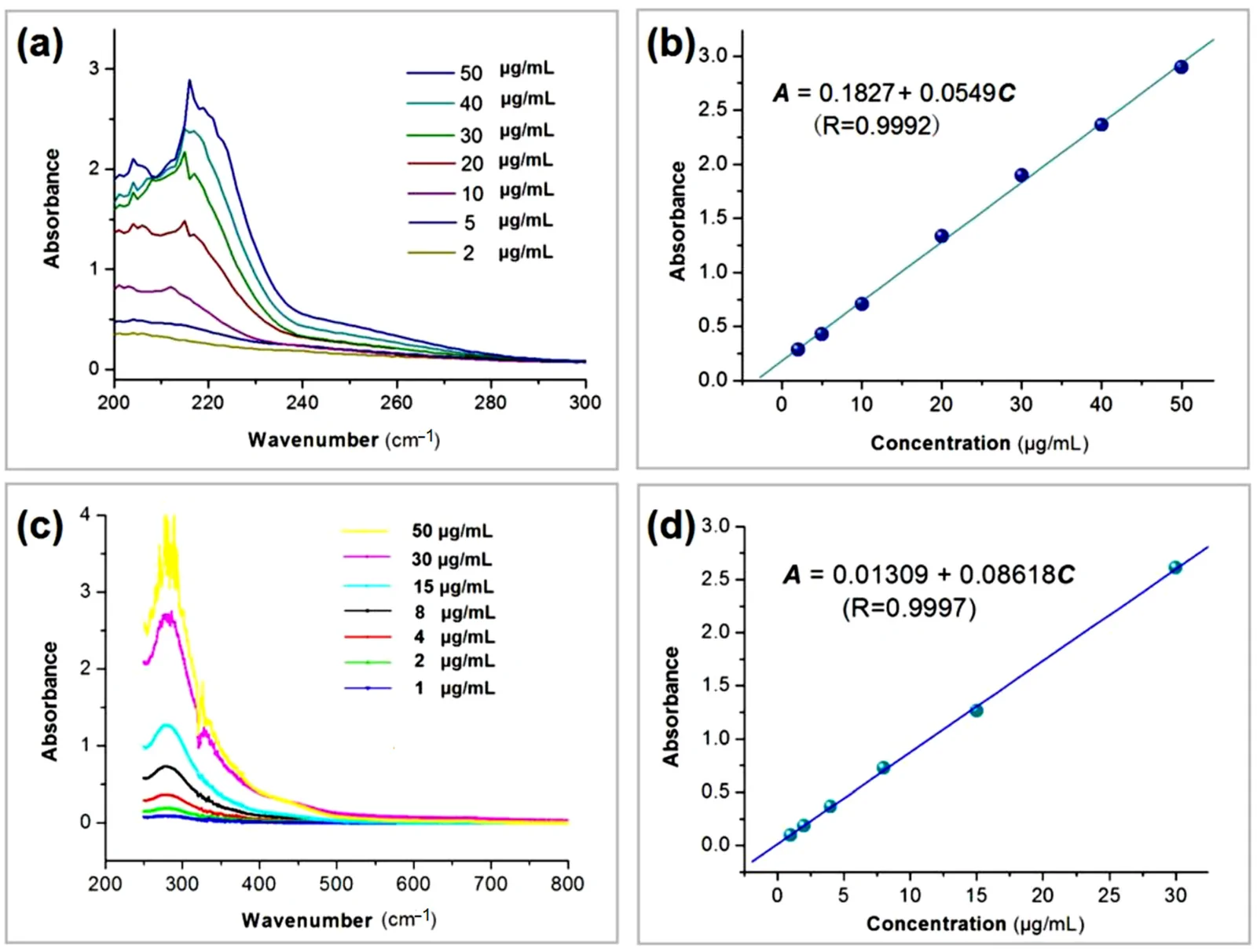
UV–vis spectrophotometric quantification of the active ingredients in JZ herbal medicine and FIN: (**a**) absorption spectra of FIN standard solutions at various concentrations; (**b**) calibration equation for FIN determination in sample solutions at λ = 220 nm; (**c**) absorption spectra of JZ herbal medicine standard solutions at various concentrations; and (**d**) calibration equation for JZ determination in sample solutions at λ = 281 nm.

Both JZ herbal medicine and FIN have obvious absorbances in the ultraviolet regions. As shown in Figure 9a, as the increase in FIN concentrations, the scanning curves for 2 µg/mL to 50 µg/mL from 200 nm to 300 nm increased gradually. With the absorbance values at the wavelength of 220 nm as the y-axis data and the FIN concentrations as the x-axis data, the regressed line is exhibited in Figure 9b. Apparently, there is a linear relationship between FIN concentration *C* (µg/mL) and its absorbance value *A* (a.u.) of *A* = 0.1827 + 0.0549 *C* with a correlation coefficient R of 0.9992 (linear range: 2 to 50 µg/mL).

Similarly, one or more of the JZ herbal medicine components have an obvious absorbance at a wavelength of 281 nm. The scanning lines of a series of standard solutions of JZ herbal medicine with a certain concentration are exhibited in Figure 9c. The linear relationship between JZ concentration C (µg/mL) and absorbance value *A* (a.u.) is *A* = 0.01309 + 0.08618 *C* with a correlation coefficient R of 0.9997 and a linear range from 1 to 30 µg/mL. Being a surrogate determination rather than a specific quantification of all JZ components, this assay warrants further investigation into more specific and validated quantification methods.

The DEE% of JZ herbal medicine in nanofibers F1 (one gram of fibers containing 169.82 ± 1.38 mg JZ) and F3-JZ (one gram of fibers containing 60.74 ± 0.31 mg JZ herbal medicine) were 93.40 ± 0.76% and 94.15 ± 0.48%, respectively. The DEE% of FIN in nanofibers F2 (one gram of fibers containing 45.38 ± 0.27 mg FIN) and F3-FIN (one gram of fibers containing 31.08 ± 1.52 mg FIN) were 95.29 ± 0.58% and 96.34 ± 0.47%, respectively. The DEE% of both JZ herbal medicine and FIN in the monolithic nanofibers F1 and F2 and the one-drug Janus nanofibrous controls F3-JZ and F3-FIN had no significant differences (*p* values were 0.1488 and 0.1420, respectively). Electrospinning is essentially a physical drying process, which is finished in an extremely fast way, leaving almost no time for the molecules in the working fluids to escape from the fluids to the environments. This should be the reason that many publications reported a DEE% value of around 100%, which is also a significant merit of electrospinning when compared with other nanofabrication methods.

**Figure 10:**
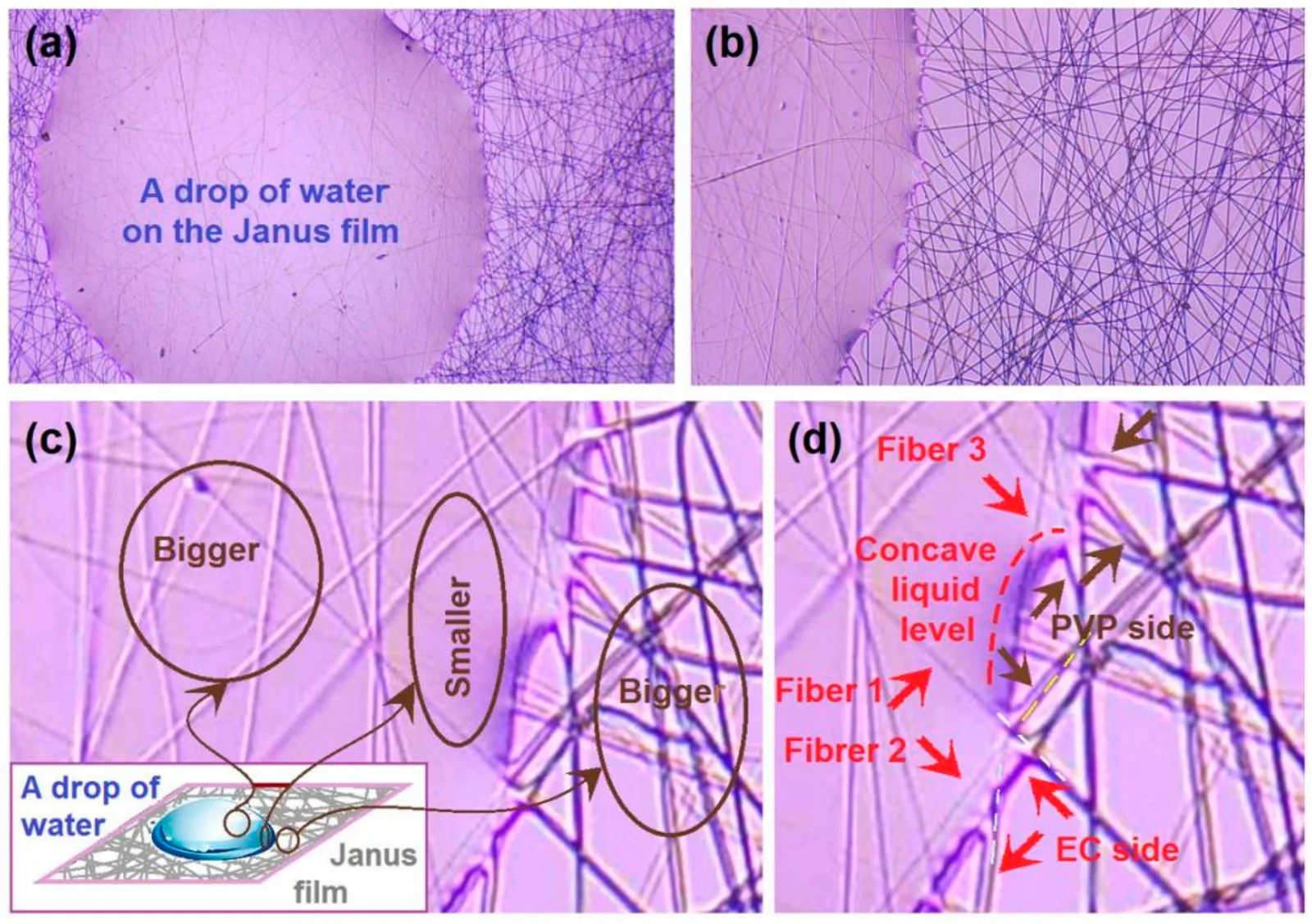
Direct observations of the fast dissolution of the PVP-JZ sample are shown in (**a**–**d**), at magnifications of 40×, 100×, 400× and 400×, respectively. The bottom-right inset of (**c**) is a schematic of a ~3 µL water droplet on the nanofibrous film collected on a glass slide.

When a drop of water with a volume of about 3 µL was dripped on the electrospun nanofibrous film collected on the glass slide, the water was rapidly expanded. The captured images with a magnification of 40 and 100 times are shown in Figure 10a and Figure 10b, respectively. The video is shown in the Supplementary Video S1. Further enlargements of the boundaries between the droplet of water and the nanofibers are exhibited in Figure 10c. An intriguing phenomenon was observed in which the apparent diameter of the nanofibers first decreased from large to small, and subsequently underwent a second large-to-small transition. As illustrated in the bottom-left inset, the initially enlarged appearance of the left-hand fibers was caused by the refraction effect of light through the water droplet. As the droplet thinned, this refractive distortion diminished, allowing the nanofibrous residues—the linear EC-FIN sides—to return to their original morphology and dimensions. In the right-hand region, the fiber sections adjacent to the water droplet became swollen due to the presence of the PVP-JZ sides; these Janus nanofibers subsequently retained their original solid state because few water molecules could penetrate there. The unnoticed swelling of EC-FIN nanofibers F2 and the obvious swelling-dissolution of PVP-JZ nanofibers F1 are included in Figure S2 of the Supplementary Materials.

A further analysis of the details on the boundaries (Figure 10d) provides a series of useful information about the nanostructures and structure–performance relationships in a qualitative manner. First of all, the Janus inner structures can be clearly demonstrated by the three nanofibers, i.e., Fiber 1, Fiber 2, and Fiber 3. The sides within the drop of water were EC-FIN, as indicated by the three red arrows pointing to the right direction. The PVP-JZ sides were completely dissolved within the droplet. Secondly, the regions indicated by the four brown arrows correspond to the PVP-JZ sides of the Janus nanofibers. Notably, the sections proximal to the droplets are substantially larger than their counterparts in the solid nanofibers, exhibiting characteristic triangular morphologies. These triangular shapes provide direct evidence of PVP swelling prior to dissolution. Thirdly, the concave meniscus observed at the right boundary, formed by several nanofibers, can be attributed to the following mechanism: the vertical EC-FIN side of Janus Fiber 3, together with several adjacent Janus nanofibers including Fiber 1, facilitated water transport across the EC-FIN side of Fiber 3. In contrast, the two red arrows pointing leftward indicate straight boundaries, which result from the hydrophobic nature of the EC-FIN sides. These detailed observations not only confirm the typical dissolution mechanism of PVP molecules—encompassing water absorption, swelling, disentanglement, and complete dissolution—but also demonstrate that the PVP-JZ sides are capable of rapid dissolution to achieve the prompt release of the encapsulated JZ herbal medicine.

**Figure 11:**
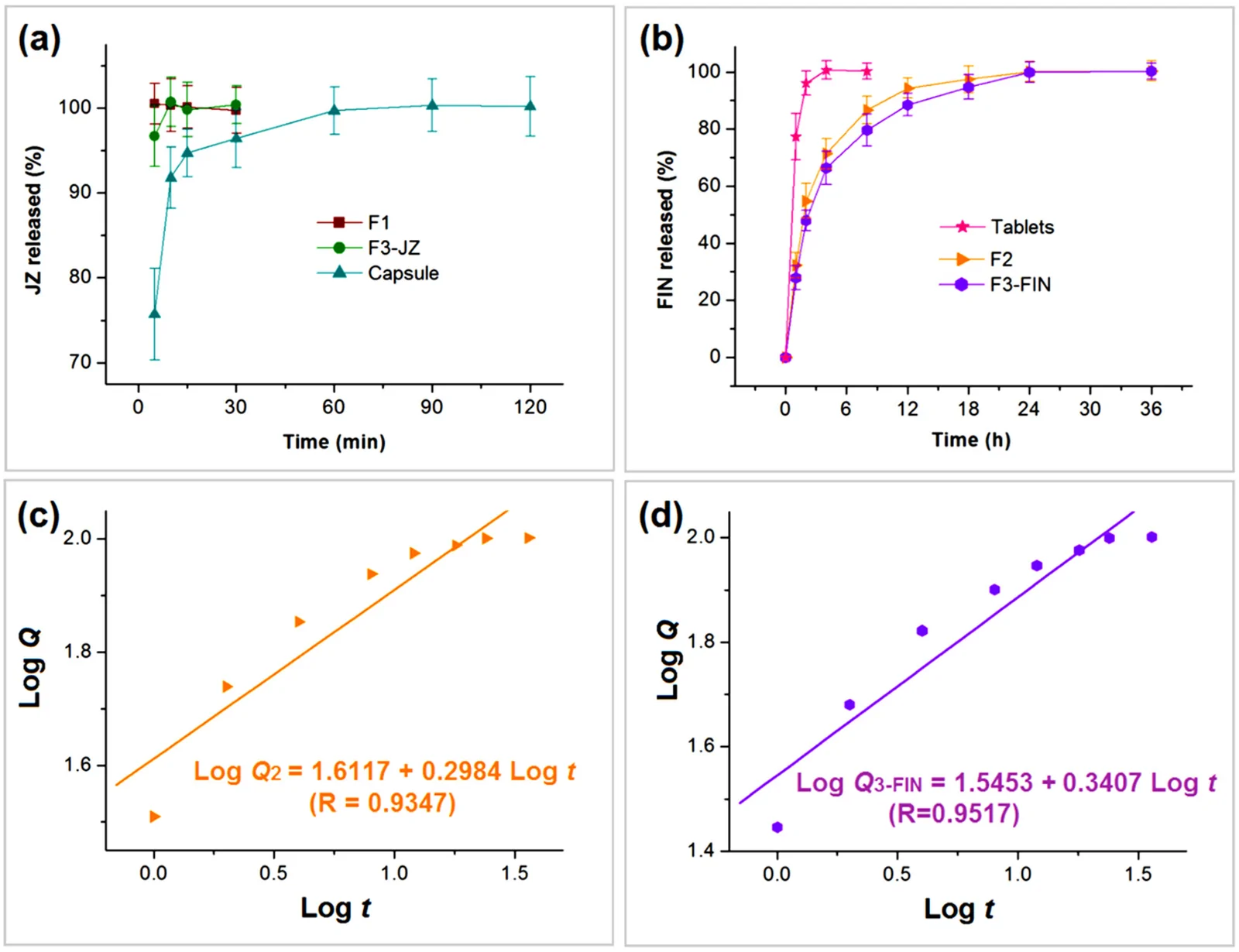
The in vitro drug release profiles of the prepared nanofibers F1 to F3 and also the controls of commercial products: (**a**) release profiles of JZ herbal medicine from the nanofibers F1, F3-JZ, and the commercial capsules; (**b**) release profiles of FIN from the nanofibers F2, F3-FIN, and the commercial tablets; (**c**,**d**) the sustained release mechanisms of FIN from the nanofibers F2 and the one-drug Janus nanofibrous controls F3-FIN, respectively.

As expected, both nanofibers F1 and the nanofibers F3-ZJ were able to release all the encapsulated JZ herbal medicine within 10 min. The commercial capsules were able to dissolve out 97.8 ± 3.6% of the components (Figure 11a). Electrospun nanofibers, having smaller diameters, large porosity, and soluble matrix, were able to free out all the components all at once. In contrast, the commercial tablets, nanofibers F2 and F3-FIN were able to provide the typical sustained release profiles. In contrast, nanofibers F2 and F3-FIN had a similar sustained release profile, and had a more prolonged release than the commercial tablets (Figure 11b). The Peppas equation is exploited to treat the sustained release data of nanofibers F2 and F3-FIN, whose results are shown in Figure 11c and Figure 11d, respectively. The regressed equation is Log *Q*_2_ = 1.6117 + 0.2984 Log *t* for nanofibers F2, and the equation is Log *Q*_3-FIN_ = 1.5453 + 0.3407 Log *t*. The exponents of nanofibers F2 and F3-FIN are 0.2984 and 0.3407, smaller than the critical value of 0.45 [63], indicating that FIN was released from the EC matrix through a typical Fickian diffusion mechanism. Many previous investigations have demonstrated that the drugs loaded into the insoluble EC matrix were released through the diffusion mechanisms [64].

In this nano era, nanostructures, nanodevices and nanoarchitectures represent one of the most important directions for nano fabrication [65,66,67,68,69,70]. Janus structure (representing a side-by-side spatial relationship in the front-back, left-right or up- down manner) and core–sheath (or core–shell, representing an inner-outer spatial relationship) comprise the most fundamental 2-chamber configurations and support one of the most powerful platforms for developing numerous functional materials. Although Janus nanostructures are popular in many fields, electrospun Janus nanofibers are extremely limited due to fabrication difficulties, an inevitable result of the electrical repulsion of the double working fluids. The present protocols provide a systematic strategy for producing Janus nanofibers with integrated structures and high quality. However, the yield is another inevitable obstacle that needs to be overcome for potential commercial drug delivery systems. Although some commercial electrospun medicated nanofibrous products can be achieved [71], and large-scale production continues to attract significant efforts, they almost exclusively consist of monolithic nanofibers from blended electrospinning [72,73,74]. Most recently, Yu et al. developed a new concept electrospinning technique for batch production of nanofibers, which is an integrated method of the traditional needle-based electrospinning and free surface electrospinning [75,76,77]. Based on the combination of the idea verified here and the new concept electrospinning method, large-scale production of the Janus nanofibers reported in this article is under investigation. By the way, during the electrospinning process, organic solvents including DCM, ethanol, and DMAc were used for material conversion. Thus, the residual solvents in the electrospun nanofibers pose a concern for the safety of their application. This issue will be exacerbated when electrospun nanofibers are produced on a large scale for commercial applications.

## 4. Conclusions

In the present study, the traditional Tibetan JZ herbal medicine and the Western small-molecule medicine FIN were co-loaded into a Janus nanostructure with each having its own controlled release profiles. A new tri-fluid electrospinning technique was successfully developed for a continuous and robust preparation of the integrated Janus nanofibers. The new tri-fluid-modified electrospinning apparatus is characterized by a new spinneret, which can ensure a co-solvent on the side-by-side parallel fluids for preventing the negative separation of double working fluids due to the mutual repelling of the same electrical charges. The resultant Janus nanofibers had the JZ herbal medicine loaded in the soluble polymeric PVP sides for a fast release, and had the FIN loaded in the inert and insoluble EC sides for a sustained release.

Both JZ herbal medicine and FIN are commercially available in capsule and tablet dosage forms and are prescribed by clinicians. In vitro dissolution tests demonstrated that the Janus nanofibrous films had potential for co-delivery of both drugs with improved release profiles. Further animal experiments and preclinical tests will be conducted. The novel nanopharmaceutical technique of modified tri-fluid electrospinning will serve as a useful tool for developing new drug-loaded nanomaterials, and the electrospun Janus nanostructures can be exploited as a useful platform for combination therapies of various diseases, particularly the possible joint effects of herbal and Western medicines.

## 5. Patents

Yu, D.-G.; Wang, X.; Li, X.; Xu, Y.; Yang, C. An eccentric spinneret and its application. Chinese Patent CN104611773A, 4 January 2017. (In Chinese).

## Figures and Tables

**Table 1:** Experimental parameters for implementing electrospinning processes.

No.	Modified Electrospinning	Working Fluid/Flow Rate (mL/h)			Operational Parameters
		Shell	Core I	Core II	
F1	2-fluid	Ethanol/0.5	Fluid 1/2.0	----	Applied voltage: 14 kV Deposition distance: 20 cm Ambient temperature: 22 ± 4 °C Relative humidity: 47 ± 7%
F2			----	Fluid 2/2.0	
F3	3-fluid		Fluid 1/1.0	Fluid 2/1.0	
F3-JZ			Fluid 1/1.0	Fluid 3/1.0	
F3-FIN			Fluid 4/1.0	Fluid 2/1.0	

Fluid 1: PVP solution comprised 9.0 g PVP and 2.0 g JZ herbal medicine in 100 mL ethanol/DMAc mixture (90:10 in volume); Fluid 2: EC solution consisted of 20.0 g EC and 1.0 g FIN in 100 mL ethanol/DCM mixture (50:50 in volume); Fluid 3: EC solution consisted of 20.0 g EC in 100 mL ethanol/DCM mixture (50:50 in volume); Fluid 4: PVP solution comprised 9.0 g PVP in 100 mL ethanol/DMAc mixture (90:10 in volume).

## Data Availability

Data presented in this study is contained within the article and Supplementary Materials. Further inquiries can be directed to the corresponding author.

## Supplementary Materials

The following supporting information can be downloaded at https://www.mdpi.com/article/10.3390/pharmaceutics18091160/s1: (1) Supplementary Materials about the commercial FIN and JZ products; the optical images of nanofibers F1, F2, F3, F3-JZ and F3-FIN; and the responses of nanofibers F1 and F2 upon contact with water; (2) Supplementary Video S1 about a droplet of water on the surface of collected Janus nanofibers on a glass slide.
