# Supplementary material for: Electrospun Integrated Janus Nanofibers for Asynchronous Delivery of Finasteride and Tibetan Medicine
Source: Pharmaceutics. 2026 Sep 15;18(9):1160. doi: 10.3390/pharmaceutics18091160 (PMC13610661; doi:10.3390/pharmaceutics18091160)
Supplement: Supplementary file 1 [file pharmaceutics-18-01160-s001.zip › Supplementary Materials.pdf]

Supplementary Materials

Electrospun Integrated Janus Nanofibers for Asynchronous Delivery of Finasteride and Tibetan Medicine

Qilin Wang, Tingyu Chen, Hailong Dou, Deng-Guang Yu*

School of Health Science and Engineering, University of Shanghai for Science and Technology, Shanghai 200093, China.

* Correspondence:
Email: ydg017@usst.edu.cn

Section S1: Commercial product of Jing-Zhu Tibetan Herbal Medicine

Jing-Zhu Qianlie Longbitong Jiaonang (Qinghai Jingzhu Tibetan Medicine High&New-tech Industry Co. Ltd., Xining, Qinghai Province.Lot No.: GUOYAOZHUNZI Z20025304), executed standard: In kidney volume of Collection of National Standards of Chinese Medicine: WS-10251(ZD-0251)-2002-2012Z.

Components: astragalusroot, eupolyphage sinensis, clustermallow fruit, peach kemel, cassia twig, epimeddiun, bupleurumroot, Tuckahoe, giant knotweed, fruit of citron or trifoliateorange, and eyathula.

Properties: This medicine is in the preparation of capsule withcontents of hoar powder of bitter and slight salt in flavor.

Functions and indications: Invigorate qi and warmyang, activate blood and promote diuresis. It could be used to treat difficulty in urination due to insufficient kidney and static blood with symptoms such as frequent urination, dysuresia, drippingurine, and weakness in the loins and knees.It could also be used to treat hyperplasia of prostate with symptoms as mentioned above.

Name of the article:
Nomenclature: Qianlie Dysuria Capsule
Chinese Pinyin: Qianlie Longbiting Jiaonang
Components: astragalus root, eupolygale sinensis, cluster mallow fruit, peach kernel, cassia twig, epimedium, bupleurum root, Tuckahoe, giant knotweed, fruit of citron or trifoliate orange, and cyathula.
Properties: This medicine is in the preparation of capsule with contents of hoar powder of bitter and slight salt in flavor.
Functions and indications: Invigorate qi and warm yang, activate blood and promote diuresis. It could be used to treat difficulty in urination due to insufficient kidney and static blood with symptoms such as frequent urination, dysuria, dripping urine, and weakness in the loins and knees. It could also be used to treat hyperplasia of prostate with symptoms as mentioned above.
Specification: 0.5g per capsule.
Usage and dosage: Taken orally, 4 capsules each time, t.i.d.. Bad response: Unknown.

Contraindication: Unknown.
Precaution: Unknown.
Storage: Stored in airtight.
Package: In blister packs, 10 capsules per packet. Expiration date: 36 months.
Executed standard: In kidney volume of Collection of National Standards of Chinese Medicine. WS-10251(ZD-0251)-2002-2012Z
Lot No.: GUOYAOZHUNZI Z20025304 www.jingzhuzangyao.com

Name of Marketing Authorization Holder and Manufacturer: Qinghai Jingzhu Tibet Medicine High & New-tech Industry Co. Ltd.
Address of Marketing Authorization Holder and Manufacturer: 36 Jingsan Road, Qinghai Biotechnology Industrial Park
Zip Code: 810003
Phone: (0971) 6315901
After-sales consultation telephone: (010) 67091268/69
E-mail: jzxs@jingzhuzangyao.com
Website: www.jingzhuzangyao.com
Any question, please contact the manufacturer freely.

Section S2: Commercial Tablets of Finasteride

Finasteride Tablets Package Insert (Simplified Version)

This package insert is a simplified version. For the most current and complete information, please refer to the electronic version (available by scanning the QR code in the upper left corner via Alipay or WeChat).

Caution for athletes.

[Drug Name]

Generic name: Finasteride Tablets

Brand name: Aishilie

[Ingredients]

Active ingredient: Finasteride

Excipients: Sodium carboxymethyl starch, lactose monohydrate, docusate sodium, pregelatinized starch, hypromellose, microcrystalline cellulose, magnesium stearate, film coating premix (gastric soluble type).

[Properties] This product is a film-coated tablet. After removing the coating, it appears white or off-white.

[Indications]

This product is indicated for the treatment and control of benign prostatic hyperplasia (BPH) and for the prevention of urologic events:

-- Reducing the risk of acute urinary retention.

-- Reducing the risk of needing transurethral resection of the prostate (TURP) and prostatectomy.

This product can reduce the size of an enlarged prostate, improve urine flow, and improve symptoms associated with prostate enlargement. It is indicated for the treatment of patients with an enlarged prostate.

[Specification] 5 mg

[Usage and Dosage]

The recommended dose is one tablet daily, each tablet containing 5 mg, taken with or without food.

Dosage in patients with renal insufficiency

No dosage adjustment is required for patients with various degrees of renal insufficiency (with creatinine clearance rates as low as 9 mL/min), as pharmacokinetic studies have confirmed that there is no alteration in the in vivo processing of finasteride.

Dosage in elderly patients

Although pharmacokinetic studies have shown a decrease in the clearance of finasteride in patients over 70 years of age, no dosage adjustment is required.

[Contraindications]

This product is not intended for use in women and children.

This product is contraindicated in the following conditions:

Hypersensitivity to any ingredient of this product.

Pregnancy — in women who are pregnant or who may become pregnant (see Use in Pregnancy and Lactation).

[Storage] Protect from light, keep tightly sealed, and store below 30°C.

[Marketing Authorization Holder]

Name: Hangzhou CONBA Pharmaceutical Co., Ltd.

Telephone and Fax: 0571-86622535 / 0571-87774127

If you have any questions, please contact the Marketing Authorization Holder.

12-231202

Section S3: Optimizations of the electrospinning processes and the typical images of the electrospun nanofibers under optical microscope

The strategy for implementing all kinds of electrospinning is the following procedure: (1) optimizing the electrospinning processes using an optical microscope; (2) Camera and also smart phone are utilized to directly observe the working process with a strong reverse light; (3) glass slides are utilized to collect nanofibers from different places on the collector; (4) when the collected nanofibers from various places are straight lines without discerned beads or spindles, the samples are prepared for the later various characterizations.

Typical optical images of the nanofibers F1, F2, F3, F3-FIN and F3-JZ under a magnification of 20×20 using an optical microscope are shown as Figure S1a to S1f.

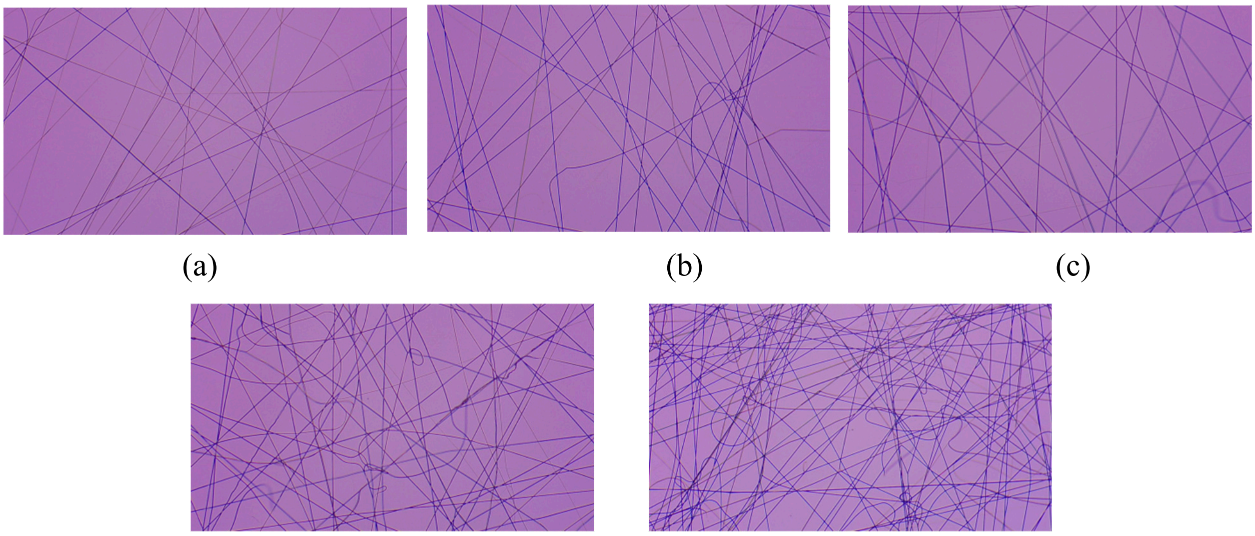

(a) (b) (c)

(d) (e)

Figure S1: Typical optical images of the nanofibers F1 (a), F2 (b), F3 (c), F3-FIN (e), and F3-JZ (f) under a magnification of ×100.

Section S4: Observations of the phenomena when a drop of water was placed on the collected monolithic PVP-JZ nanofibers F1 and EC-FIN nanofibers F2

To observe the droplet of water resting on the insoluble EC-FIN nanofibers, images of the dry nanofibers without water (Figure S2a1) and those within the droplet (Figure S2a2) were captured at various focal planes. The straight, linear EC-FIN nanofibers directly formed the boundaries of the droplet. The enlarged image in Figure S2a3 shows that none of the EC-FIN nanofibers swelled when protruding from the water.

Similar observations were made at different locations: the PVP-JZ nanofibers rapidly absorbed water, swelled, and dissolved upon contact with a droplet (Figure

S2b1 and S2b2). The image in Figure S2b3 provides a magnified view of the water absorption, swelling, and dissolution processes of PVP-JZ nanofibers upon contact with water.

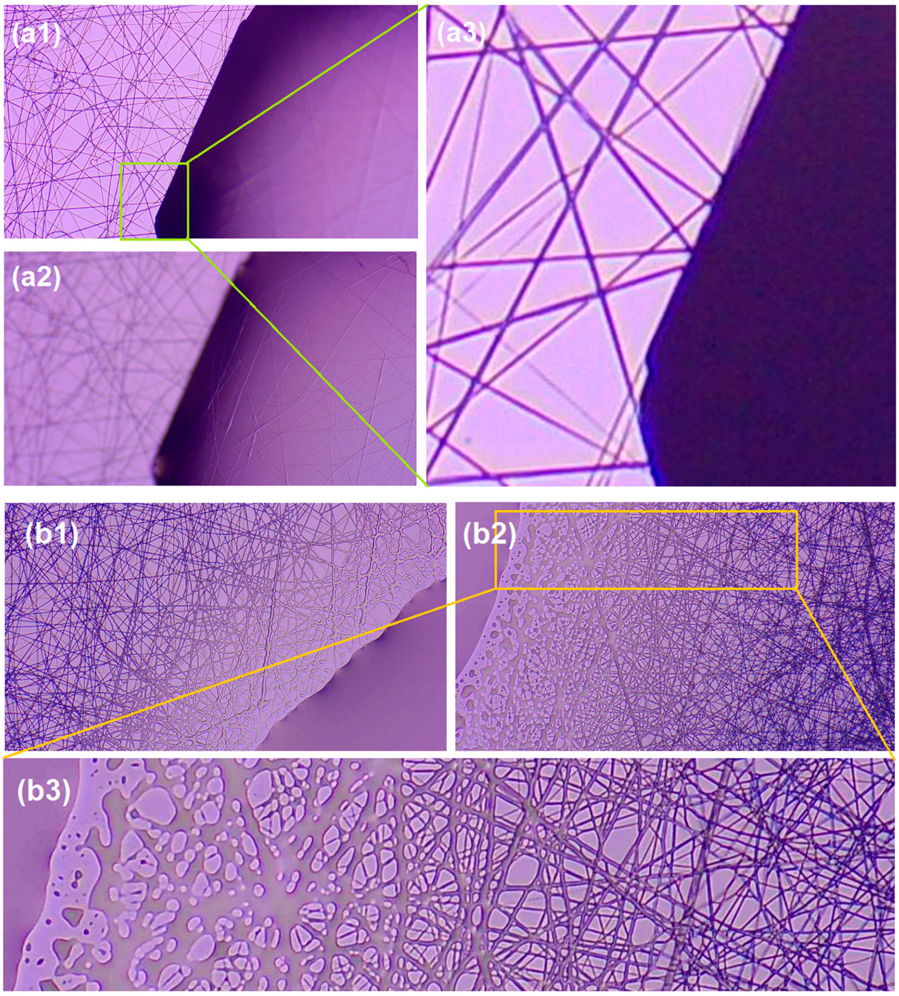

Figure S2. Typical optical images of nanofibers F1 (a) and F2 (b) after a water droplet was placed on the samples collected on glass slides. (a1 and a2) Same-field images focused on solid nanofibers outside and inside the water droplet, respectively (×100); (a3) magnified view of the droplet boundary, showing no obvious swelling of the EC-FIN nanofibers. (b1 and b2) Images from two different locations showing the transition from water droplet to swollen nanofibers to solid nanofibers (×100); (b3) magnified image showing the water absorption–swelling–dissolution sequence of PVP-JZ nanofibers upon contact with water.
